# Emerging Role of miR-345 and Its Effective Delivery as a Potential Therapeutic Candidate in Pancreatic Cancer and Other Cancers

**DOI:** 10.3390/pharmaceutics13121987

**Published:** 2021-11-23

**Authors:** Nagabhishek Sirpu Natesh, Brianna M. White, Maia M. C. Bennett, Metin Uz, Rakhee Rathnam Kalari Kandy, Surinder K. Batra, Surya K. Mallapragada, Satyanarayana Rachagani

**Affiliations:** 1 Department of Biochemistry and Molecular Biology, University of Nebraska Medical Center, Omaha, NE 68198-5870, USA; snagabhishek@unmc.edu (N.S.N.); maiabennett@unomaha.edu (M.M.C.B.); rakheerathnam@gmail.com (R.R.K.K.); sbtara@unmc.edu (S.K.B.); 2 Department of Chemical and Biological Engineering, Iowa State University, Ames, IA 50011, USA; bmwhite@iastate.edu (B.M.W.); suryakm@iastate.edu (S.K.M.); 3 Department of Chemical and Biomedical Engineering, Cleveland State University, Cleveland, OH 44115-2214, USA; m.uz@csuohio.edu; 4 Fred & Pamela Buffet Cancer Center, Eppley Institute for Research in Cancer and Allied Diseases, University of Nebraska Medical Center, Omaha, NE 68198-5950, USA

**Keywords:** PDAC, miR-345, miRNA nanodelivery

## Abstract

Pancreatic ductal adenocarcinoma (PDAC) is an aggressive malignancy with high mortality, poor prognosis, and palliative treatments, due to the rapid upregulation of alternative compensatory pathways and desmoplastic reaction. miRNAs, small non-coding RNAs, have been recently identified as key players regulating cancer pathogenesis. Dysregulated miRNAs are associated with molecular pathways involved in tumor development, metastasis, and chemoresistance in PDAC, as well as other cancers. Targeted treatment strategies that alter miRNA levels in cancers have promising potential as therapeutic interventions. miRNA-345 (miR-345) plays a critical role in tumor suppression and is differentially expressed in various cancers, including pancreatic cancer (PC). The underlying mechanism(s) and delivery strategies of miR-345 have been investigated by us previously. Here, we summarize the potential therapeutic roles of miR-345 in different cancers, with emphasis on PDAC, for miRNA drug discovery, development, status, and implications. Further, we focus on miRNA nanodelivery system(s), based on different materials and nanoformulations, specifically for the delivery of miR-345.

## 1. Pancreatic Cancer

Cancer is one of the leading causes of death globally, acting as a crucial barrier to enhancing life expectancy, with 19.3 million worldwide new cancer incidence accounts and 10 million cancer deaths in 2020 [1]. Pancreatic cancer (PC) is the fourth-highest cause of cancer mortality, with an overall 10-year survival of less than 1% and 5-year survival rate of less than 3%, based on statistics of patients from England and Wales [2,3]. In 2020, PC accounted for 0.49 million (2.6%) new cases and 0.46 million deaths (4.7%) worldwide [1]. PC accounts for about 3% of all cancers and about 7% of all cancer deaths in the USA [4]. A study of 28 European countries showed that PC would surpass breast cancer as the third leading cause of cancer deaths by 2025 [5].

Pancreatic ductal adenocarcinoma (PDAC) neoplasms that arise from the duct of the exocrine pancreas account for >90% of PCs and contribute to major medical complications. In contrast, PCs that arise from the endocrine gland (pancreatic neuroendocrine tumors (NETs)) are less common (<5%). It is estimated that 1 in 64 individuals will develop PDAC, an age-related neoplasm, with an average age at diagnosis of about 71 yrs [6]. With many developed countries facing aging populations, PC incidence level is expected to rise over the coming years and be the second leading cause of death in the US by 2030 [7,8]. PDAC is an aggressive disease that invades the lungs, liver, peritoneal cavity, lymph nodes, and intestines [9]. Symptoms including abdominal pain, weight loss, bloating, nausea and vomiting, new-onset diabetes, back pain, shoulder pain, pruritus, dyspepsia, lethargy, jaundice, and changes in bowel habits, developed during PDAC, are often neglected by the patients [10].

The modifiable risk factors associated with PC are (i) smoking, with a strong association of 74% increased risk in current smokers and a 20% increased risk in former smokers; (ii) alcohol, with a 15–43% increased risk based on meta-analysis; (iii) obesity, with a 10% increased risk for every 5 BMI units (body mass index); (iv) dietary factors, with a non-significant positive association for red meat and a 17% increased risk associated with 50 g/d of processed meat consumption, compared to 20 g/d; and (v) the presence of *Helicobacter pylori*, with a 45% increased risk [11]. Several DNA mismatch repair genes, which include MLH1, MSH2, MSH6, and PMS2, have been reported to be associated with an increased incidence of PDAC [12]. Familial atypical multiple moles-melanoma syndromes (CDKN2A gene mutation), and hereditary pancreatitis (PRSS1 and SPINKI genetic abnormalities) are also associated with an increased risk of PDAC [13,14]. There is also an immense increased risk of PDAC associated with germline mutation of ovarian and breast cancers (PALB2 and BRCA1/BRCA2, respectively), as alterations in germline *BRCA* and *PALB2* are detected in approximately 5–9% of patients with PDAC and can lead to homologous repair deficiency (HRD) [15].

Although substantial progress has been made in developing many novel cancer therapies, PC survival rates have been static in the last 4 decades, and cytotoxic therapies and their combination with targeted therapies for cancer-associated molecular pathways have not shown adequate results [2]. This is mainly due to (1) the asymptomatic nature of PDAC, (2) lack of specific tumor markers and late-stage diagnosis of the disease, (3) the retroperitoneal position of the pancreas, which limits the imaging capacity to detect this disease at its early stages, (4) PDAC’s high chemotherapy resistance and broad heterogeneity of genetic mutations, (5) a dense stromal environment, (6) rapid upregulation of alternative compensatory pathways, and (7) a majority of patients with unresectable, locally advanced, or metastatic disease at the time of diagnosis [16,17,18], contributing to poor prognosis and dismal surval rates. These factors indicate that if PC is diagnosed earlier, there is a significantly improved chance of disease control with effective treatment [2]. More than two-thirds of PC cases found are associated with diabetes mellitus (DM), linked with increased metastasis in PC patients and an increased inflammatory response, with high glucose levels stimulating PC progression [19]. Previous reports have also shown an increased risk of PC in type 2 DM patients [20].

Current available therapeutic options for PC are surgery, radiation, chemotherapy, immunotherapy, and targeted therapy [21]. Most of the available treatments are palliative, to relieve disease-related symptoms and prolong survival. Recently, targeting major cancer pathways using microRNAs (miRNAs) has shown a potential therapeutic option for cancer treatment due to their ability target more than 200 genes, as reported in our previous studies [22,23]. Thus, inhibition (oncomiRs) or replacement (tumor suppressors) of microRNAs is a promising area of study for therapeutics. PC has several challenges, in terms of diagnosis, treatment, and prognostic outlook. This review will explore how miR-345 and its effective delivery can play a significant role in disease screening, prognoses, and new therapeutic options.

## 2. PC and miRNA

There is an urgent need to identify novel diagnostic/therapeutic targets for the effective management of highly malignant PC [18]. miRNAs have received significant attention as a new class of non-coding-RNAs engaged in the regulation of gene expression. It is now evident that miRNAs exhibit differential expression in cancer and play essential roles in the disease processes [24]. Biosynthesis of miRNA is a multi-step process, which involves both cytoplasmic and nuclear components. First, RNA polymerase II transcribes miRNA into longer primary transcripts (pri-mRNA) of several hundred kilobases in length. These transcripts have 5′ 7-methyl guanylate (m7G) cap structure and 3′ poly(A) tail, and two mature endonuclease reactions (in the nucleus by Drosha/DGCR8, a ribonuclease III (RNase III) endonuclease that cleaves the pri-miRNA into a 65–70 bp stem-loop precursor miRNA (pre-miRNA)). The pre-miRNA is then transported to the cytoplasm by an exportin-5–RanGTP-dependent mechanism. Further, in the cytoplasm, the pre-miRNA is processed to generate mature 19–24 bp RNA duplex by RNase III endonuclease, Dicer/TRBP, and AGO2. Then, this RNA duplex unwinds and matures to a single-stranded miRNA (one of the strands) by the helicase activity. Further, it enters into the RNA-induced silencing complex (RISC) and directs the complex to target mRNA, through a poorly defined mechanism. It is extremely rare to have a perfect base pair binding of miRNA and the genes, but a strong base-pairing between the 5′ half of the miRNA (2–8 nucleotides) mediates the mRNA regulation in animals [25]. Any complementarity between the seed region of miRNA and 3′ UTR region of the target gene leads to translation inhibition. Hence, one miRNA is predicted to target more than 200 genes, on average coordinating multiple pathways, and regulating the critical processes of gene activation and suppression. This diverse role leads to the complexity of gene regulation, thus mediating complex cell function networks, such as cell proliferation, differentiation, apoptosis, and organ development [26].

Different patterns have been found in miRNA expression profiles in PC, which have contributed to the development of a miRNAome between the normal and cancerous pancreas [2]. Determination of these miRNA expression profiles has been made possible through different gene profiling methods, mainly microarray, RNA-sequencing, and RT-PCR analysis of specimens [27]. Due to the stability of miRNA in circulation, blood screening could be employed to detect specific miRNA linked with stage, survival rate, or aggressiveness of the disease [28]. Previous studies have demonstrated the potential of miR-483-3p and miR-21 as biomarkers of PDAC from blood plasma. The plasma expression of both miR-483-3p and miR-21 was found to be significantly higher in PDAC patients, compared to healthy controls (*p* < 0.01), while these miRNA expression levels correlated with overall lower survival in those patients with PDAC (*p* < 0.01) [29]. Indeed, miR-375 was suggested to be linked with islet cells, as the expression was high in normal pancreatic tissues, compared to the cancerous and inflammatory tissues, with a complete absence in representative cell lines. The serum carbohydrate antigen 19-9 (CA-19-9) been employed as a marker for assessing clinical treatment efficacy in PC. Limitations associated with CA 19–9 include ineffectiveness, low sensitivity, and specificity, yet it is still the only FDA approved marker in PC [29]. miRNAs that are specific to cancer types can be identified for early screening, and miRNAs could be used for diagnostic purposes, as they are stable in serum, ease the non-invasive detection in circulation, and provide a convenient screening method for miRNA detection. Among the top aberrantly expressed miRNAs in cancer samples, miR-424, miR-100, miR-301, miR-212, and miR-125b-1 were overexpressed, whereas miR-345, miR-142-P, and miR-139 were underexpressed, relative to normal pancreatic samples. Additionally, miR-221, miR-376a, and miR-301 were found to be localized within the tumor cells, rather than other cells in the stroma [30].

The miRNAs miR-141, miR-148a, miR-200a, miR-200b, miR-200c, miR-216, miR-217, and miR-375 exhibited high expression levels, and miR-133a, miR-143, miR-145, and miR-150 exhibited low expression levels within normal pancreatic tissue, compared to 33 other human tissues [31]. Upregulated miR-372, miR-146a, miR-204, miR-10a, and miR-10b were also detected in PC cell lines (CAPAN-1 and CFPAC-1), compared to human normal pancreatic ductal epithelial cells (HPDE), with changes of greater then 10-fold. miR-93, miR-196a, miR-196b, miR-203, miR-205, miR-210, miR-221, miR-222, and miR-224 were upregulated only in cancerous tissues and cell lines. Interestingly, a complete absence of miR-196a and miR-196b was observed in normal and pancreatitis tissues [32]. This gives a potential selectivity to PC. The upregulation of miR-155, miR-203, miR-210, and miR-222, as well as the downregulation of miR-216 and miR-217, are associated with poorer prognosis and overall survival [33]. Among these miRNAs, miR-345 has been gaining attention, due to its involvement in mediating signaling pathways and crosstalk in PDAC and PC, as well as other cancer types. Therefore, the following section will focus on the implications of miR-345 in multiple human malignancies, where PDAC would be given special emphasis.

## 3. Role of miR-345 in Pancreatic Cancer

miR-345 is a small, non-coding RNA, located at human chromosome 14q32.2, that is reported to have dynamic roles in various human pathologies. Differential expression of miR-345 has been reported in the blood and tissues of multiple human malignancies, such as pancreatic ductal adenocarcinoma (PDAC), gastric cancer (GC), colorectal carcinoma (CRC), prostate cancer (PCa), and breast cancer (BRCA) [33]. This differential regulation of miR-345 has been correlated with excessive cell proliferation, invasion, and migration, which are characteristics of cancer development and metastasis. Recent research has gained much attention, implicating the role of miR-345 in various human pathologies [34].

**Figure 1:**
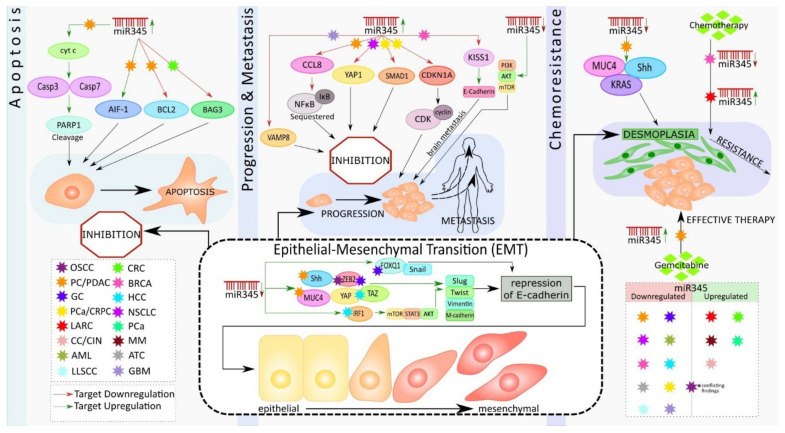
Schematic representation of the role of miR-345 in different cancer types.

Epithelial-mesenchymal transition (EMT) is a biological switch that involves the transdifferentiation of epithelial cells attached to the basement membrane and is characterized by apical-basal polarity. Increased mesenchymal phenotypes with spindle-shaped appearance, invasiveness, and enhanced migratory capacity ultimately lead to the demolition of the basement membrane [33]. EMTs occur during various biological processes and are classified into three types: the first type occurs during embryonic development; the second type is associated with adult tissue regeneration; and the third type occurs in cancer progression [35]. Numerous cellular signaling pathways and their crosstalk in the regulation of transcription factors and ultimately trigger EMT in cancer [36]. miR-345 is one the most critical miRNAs that helps to mediate the metastasis signaling pathways of EMT [37]. Studies have found that miR-345 can regulate the upstream and downstream molecules of PI3K/AKT, mTOR, and YAP/TAZ signaling [38,39,40] (Figure 1).

Previously, miR-345 was identified as one of the most significantly downregulated miRNAs in PC; however, its functional significance remained unexplored. One study showed that downregulation of miR-345 was a frequent event in PC, and this downregulation significantly correlated with PC progression [41]. Further, ectopic expression of miR-345 in PC cells dramatically reduced cell growth and induced apoptosis [22].

Srivastava et al. showed that miR-345 can induce apoptosis in caspase-dependent and -independent mechanisms in PC cells. They also observed downregulated miR-345 expression in PC tissues and cell lines. They further demonstrated that the overexpression of miR-345 disrupted mitochondrial membrane potential in PC cells, resulting in the release of cytochrome C (Cyt c) to the cytosol from the mitochondria, causing the activation of Caspase 3 and 7 with subsequent cleavage of poly [ADP-ribose] polymerase 1 (PARP-1) (Figure 1). Additionally, overexpressed miR-345 induced the translocation of the apoptosis-inducing factor (AIF-1) to the nucleus, leading to caspase-independent apoptosis. Their experiments identified B-cell lymphoma 2 (BCL2), an anti-apoptotic molecule, as the direct target of miR-345. The authors considered the miR-345 downregulation-mediated upregulation of BCL2, the switch that triggered the observed apoptosis resistance in PC [42], (Figure 1).

Apoptosis is a vital tool that assists in biological events, such as tissue homeostasis, tissue development, and immunity. Dysregulation of apoptosis is often associated with tumorigenesis [43]. The primary event in apoptosis is the loss of the mitochondrial membrane potential, which ultimately results in mitochondria-mediated apoptosis [44]. Cyt C is released, in response to the changes in mitochondrial membrane potential, binding with apoptotic protease activating factor-1 (APAF-1) and ATP (adenosine triphosphate), resulting in the formation of a complex. This complex binds to pro-cas-9, which, in turn, causes the cleavage of it and activation of Cas-3 and 7 (effector caspases). Once formed, these effector caspases cleave PARP-1 and mediate caspase-dependent apoptosis [45] (Figure 1). In the case of caspase-independent apoptosis, AIF, from the inter-mitochondrial membrane space, translocates to the nucleus, which results in chromatin condensation, nuclear fragmentation, and, subsequently, cell death [46].

Previous research has shown an increase in the level of BCL2 in numerous cancer types, including PC, and is associated with apoptosis resistance and cancer metastasis [47]. BCL2 regulates the mitochondrial release of Cyt c and AIF, which favor cell survival. Thus, the downregulation of BCL2 by miR-345 holds significance in PC therapy. Tzifi et al. further detail the blockage of the Cyt c release, in response to the overexpression of BCL2, and associated abolishment of apoptosis process [48] (Figure 1).

Srivastava et al. also showed that miR-345 is differentially expressed in PC cell lines, with more downregulated expression in poorly differentiated cancer cells. Ectopic overexpression of miR-345, in two poorly differentiated PC cell lines, Panc1 and MiaPaCa, diminished their growth, clonogenicity, motility, and invasion and enhanced cell–cell interaction. miR-345-overexpressing cells had distinguishable morphological differences and increased epithelial marker (E-cadherin) expression, as well as loss of mesenchymal (N-cadherin, vimentin, twist, slug, and snail) markers expression, as compared to the control cells. Further, the data showed that miR-345 overexpressing PC cells were more sensitive to gemcitabine (GEM), as compared to the control cells. Altogether, these findings demonstrate novel tumor suppressive roles of miR-345 in PC and indicate that miR-345 downregulation may be one of the mechanisms underlying the chemoresistance of PC [42] (Figure 1).

Another study by Mou et al. showed that the downregulated expression of miR-345 in PDAC tissues and cells, and the overexpression of miR-345 in PANC1 and SW1990 cell lines in vitro inhibited proliferation and metastasis by inactivating the nuclear factor κB (NFκB) signaling pathway and further suppressed PC progression [49]. Constitutive activation of NFkB signaling has been reported in approximately 70% of PDAC cases [50]. NFκB is an important transcription factor (TF) that has been widely researched, in the context of inflammation. Constitutive or induced activation of this transcription factor is the key to inflammation-driven carcinogenesis [51]. NFκB operates either by canonical or non-canonical pathways, and the activity of this TF is tightly regulated by the inhibitors of κB (IκB). These inhibitory κB molecules mask the nuclear localization signal (NLS) and thereby sequester the latent NFκB molecule in the cytoplasm [52]. Phosphorylation of IκB molecules by inhibitory κB kinase (IKK), in response to cellular stimuli, results in the ubiquitination and proteasomal degradation of IκB, allowing NFκB to enter the nucleus and regulate the target gene expressions associated with cell cycle regulation, proliferation, apoptosis, inflammation, and cancer metastasis [53]. CCL8 (Chemokine C-C motif Ligand 8/MCP2), a cytokine of CC chemokine family, interacts with numerous cell-signaling receptors, regulates and controls leukocyte chemotaxis, and attracts tumor-associated macrophages, inflammatory diseases, and HIV entry [54]. The dynamic role of CCL8 in tumor progression and invasion, by activating NFκB signaling, is widely accepted in different cancer types [55]. CCL8 has been identified as the direct target of miR-345 in PDAC. Further, the overexpression of miR-345 resulted in a low level of CCL8 that, in turn, reduced the nuclear protein level of NFκB and subsequent inhibition of proliferation and migration of PDAC cells. These results suggest that miR-345-5p potentially inhibits PDAC progression by inactivating NFκB signaling [49] (Figure 1).

Restoration of miR-345 in PC cell lines resulted in the inhibition of growth, motility, invasion, and upregulation of epithelial markers [42]. At the molecular level, the ectopic-expression of miR-345 in PC cell lines led to significant downregulation of SHH, MUC4, Kras, C-Myc, CSC, and survival markers, as well as the upregulation of cleaved caspase 7 & 3 and PARP (Figure 1). Sonic hedgehog (Shh) and MUC4 signaling pathways play an essential role in tumor growth and metastasis by promoting EMT, PC stem cells, angiogenesis, and desmoplasia, which limit the delivery and efficacy of chemotherapy [56,57,58]. Therefore, miR-345 is an excellent candidate for diagnostic/prognostic and therapeutic targets in PC.

## 4. Role of miR-345 in Other Cancer Types

### 4.1. Colon and Rectal Cancer

Schou et al. studied the role of miRNA in colorectal cancer (CRC), aiming to find miRNA expression profiles in whole blood that were prognostic for overall survival (OS) in patients with metastatic colorectal cancer (mCRC) treated with cetuximab and irinotecan. Interestingly, they found that miR-345 was the strongest prognostic miRNA, significant in the full cohort and non-KRas mutant population, as well as in progression-free survival (PFS). Thus, miR-345 in whole blood was a potential single prognostic biomarker for a clinical outcome for OS in all patients. In addition, high miR-345 expression was associated with a lack of response to treatment with cetuximab and irinotecan [59].

Another study, by Tang et al., shows how aberrant methylation of miRNAs is found to be deregulated in human CRC. They also investigated CpG island promoter hypermethylation, as a potential mechanism underlying miRNA disruption, and identified methylation-sensitive miRNAs that might repress CRC development. Comparative differential expression of miRNAs post-treatment with 5-aza-2′-deoxycitidine (5-aza-dC) was studied using microarrays, and it was found that the expression of miR-345 was significantly down-regulated in 51.6% of CRC tissues, compared with normal tissues. DNA methylation analyses of miR-345 showed high methylation levels in tumors versus normal tissues. Low expression of miR-345 was associated with lymph node metastasis and a worse histological type. Increased miR-345 function was sufficient to suppress colon cancer cell proliferation and invasiveness in vitro. Furthermore, they also demonstrated that BCL2-associated athanogene 3 (BAG3), an anti-apoptosis protein, is a target of miR-345. These results suggested that, as a methylation-sensitive miRNA in CRC, miR-345 may play an important role of antineoplastic as a growth inhibitor in CRC development [37] (Figure 1).

Few reports to date have investigated the association between circulating miRNA signatures and preoperative chemoradiotherapy (pre-CRT) pathological response in rectal carcinoma. Pre-CRT has been represented as the standard treatment for locally advanced rectal cancer (LARC), but significant variations of tumor radiation response to CRT have been reported in the clinic. Global miRNA expression was assessed in CRT-sensitive and -resistant groups and it was observed that miR-345 was significantly elevated in the CRT-resistant group. High miR-345 expression was significantly correlated with an unfavorable pre-CRT pathological response in tissue and serum, acting as a promising biomarker [60] (Figure 1).

### 4.2. Gastric Cancer

Feng et al. showed that miR-345 expression was decreased, in the case of gastric cancer (GC) cell lines (SGC-7901, AGS, MKN-45, MGC-803) and tissues. Reduced expression of miR-345 was associated with the occurrence of lymph node metastasis and advanced TNM (tumor (T), nodes (N), and metastases (M)) stages in GC patients. Low expression levels of miR-345 have shown reduced overall survival (OS) and disease-free survival (DFS) in GC patients. They also showed that miR-345 knockdown could promote lung metastasis, through induction of the EMT phenotype and by repressing the expression of E-cadherin, an epithelial marker, which resulted in an aggressive phenotype of GC cells in nude mice. In addition, they showed that miR-345 could inhibit the epithelial-mesenchymal transition of GC (Figure 1).

Further, Forehead box Q1 (FOXQ1) was confirmed to be the downstream target of miR-345 in GC cells. FOXQ1 is a member of the forehead box protein family, which functions as a transcription factor, associated with the regulation of cell differentiation and metastasis [61,62]. FOXQ1 binds to the E-box present at the promoter region of E-cadherin and represses its expression, thus resulting in EMT and its associated enhanced migratory capacity of the cells [62]. FOXQ1 could reverse the inhibitory effects of miR-345 on GC metastasis, while knockdown prevented the promoting effects of miR-345 knockdown on GC metastasis [63] (Figure 1). This study demonstrates that miR-345 is a promising biomarker and therapeutic target in GC. The functional relevance of FOXQ1 in multiple human cancers, such as gastric cancer, esophageal cancer, PDAC, carcinoma of the breast, carcinoma of lung, adenoma, and carcinoma of the intestine, has been appreciated in the recent scientific literature [61,64,65,66,67].

### 4.3. Prostate Cancer

The role of miR-345 in prostate cancer (PCa) was studied by Tinay et al., where they compared under expression in PCa with normal patients. They discovered that miR-345-5p were significantly overexpressed in serum from prostate cancer patients and further demonstrated that miR-345-5p (Two mature miRNA species may be generated from the 5′[-5p] and 3′[-3p] arms of a pre-miRNA precursor. In most cases, only one species remains while the complementary species is degraded. To avoid further confusion and ambiguity by prematurely presuming expression levels and biological functions, a specific miRNA strand has been represented), promotes CRPC (Castration-Resistant PCa) cell growth and migration in vitro and validated CDKN1A (the gene encoding p21) as the direct target. Thus, circulating miR-345-5p can be used as a biomarker for PCa diagnosis and therapeutic response. CDKN1A inhibits the cyclin/CDK complex that, in turn, leads to cell cycle inhibition, ultimately resulting in cancer suppression. The oncogenic roles of miR-345-5p, through targeting CDKN1A, render it a potential therapeutic target for PCa [68] (Figure 1). Another study by Chen et al. showed that overexpression of miR-345 in prostate cancer cells suppressed proliferation, migration, and invasion. Using a xenograft tumor (athymic nude mouse) model, they revealed that miR-345 inhibits the growth of prostate cancer cells both in vivo and in vitro. Furthermore, they identified and validated Smad1 as a direct target of miR-345 [69].

### 4.4. Lung Cancer

The role of miR-345 in lung cancer was studied by Zhang et al., where they showed that prominent downregulation was associated with the patient poor prognosis in non-small-cell lung carcinoma (NSCLC) tissue specimens [70]. They also found that miR-345 overexpression could dramatically inhibit NSCLC cell migration and invasion. Further, the study also identified Yes-associated protein 1 (YAP1) as the direct functional target of miR-345 in NSCLC cells, as YAP1 expressions were negatively correlated with the miR-345 in NSCLC tissue samples. Moreover, YAP1 was found to be involved in the functions of miR-345 in inhibiting NSCLC cell invasion and migration [70] (Figure 1). YAP1, an oncogenic transcriptional coactivator, has recently been identified as a molecular target for tumor therapy. YAP1 is implicated in regulating multiple cellular processes, including cell apoptosis, proliferation, growth, tumorigenesis, stem cell differentiation, and renewal. Wang et al. (2012) identified five miRNA (miR-93, miR-100, miR-134, miR-151, and miR-345) signatures significantly associated with overall survival and can predict patient survival in advanced stages of NSCLC. High expression of miR-100 and low expression of miR-93, miR-134, miR-151, and miR-345 were associated with poor survival outcomes. These findings may have implications in the understanding of advanced NSCLC [71]. Overall, miR-345 expression was downregulated in NSCLC, and its low expression was correlated with malignant clinical parameters and poor prognosis [72].

### 4.5. Liver Cancer

Jiang et al. showed that hepatocellular carcinoma (HCC) patients with good survival rates had high miR-345 expression levels, compared with expression in cases with poor survival rates [73]. Shiu et al. reported that hepatitis C virus (HCV) core protein upregulated the expression of miR-345, which inhibited curcumin-induced apoptosis by targeting p21 in Huh7 cells [74]. Another study by Yu et al. showed that miR-345 underexpression was observed in HCC tissues and cells and illustrated that the loss of miR-345 promoted HCC cell migration and invasion and resulted in epithelial-mesenchymal-transition (EMT) progression, probably by targeting interferon regulatory factor 1 (IRF1)-mediated mTOR/STAT3/AKT signaling in vitro. It was found that the mTOR/STAT3/AKT pathway and its downstream targets, including Slug, Snail, and Twist, may be involved in the IRF1-mediated EMT process (Figure 1). Their work provided the first evidence for miR-345 in prognosis and treatment in HCC [40].

Ding et al. explored the role of miRNA in liver cancer, showing that miR-101 can reduce the level of hepatitis B virus (HBV) replication and inhibit the proliferation of hepatoma cells. In addition, they also found miR-345 can upregulate the level of HBV replication and promote the proliferation of liver cancer cells [75]. Hepatitis B is a major infectious disease worldwide, chiefly caused by the HBV infection, where HBV enters hepatocytes and destroys their DNA, causing cirrhosis of the liver that, in some cases, develops into liver cancer [76,77]. A recent study by Zhang et al. (2017) showed that YAP1 had been identified as the direct functional target of miR-345 in HCC. Under-expression of miR-345 was linked with poor prognosis, with reduced miR-345 expression in HCC tissues and cell lines (MHCC-97H, HEP3B) [78] (Figure 1). miR-345 overexpression was shown to greatly reduce the migratory and invasive properties of MHCC-97H. Knockdown of miR-345 exhibited an enhanced lung metastasis of HCC cells in vivo and Hep3B cells in vitro, respectively. At the same time, overexpression and knockdown of YAP1 reversed the entire condition. YAP1 is a downstream molecule of hippo signaling, increasingly reported in the aspect of cancer progression and metastasis [79]. YAP/TAZ elevates specific genes such as CDK1, MCM, CDC25, which are involved in cellular proliferation and cell cycle [80]. Many studies demonstrated that YAP, along with the transcriptional coactivator with PDZ-binding motif (TAZ), regulates cell cycle, invasion, migration, and EMT [81] (Figure 1).

### 4.6. Cervical Cancer

miR-345 was upregulated in cervical cancer (CC) and studied by Cheung et al. (Figure 1). They studied the distinct miRNA expression signatures for cervical intraepithelial neoplasia (CIN) and normal cervical epithelium. Out of the 202 miRNAs evaluated, 12 were highly differentially regulated miRNAs, including miR-518a, miR-34b, miR-34c, miR-20b, miR-338, miR-9, miR-512-5p, miR-424, miR-345, miR-10a, miR-193b, and miR-203. Seven of these twelve miRNAs (miR-9, miR-20b, miR-345, miR-338, miR-518a, and miR-512-5p) were upregulated, and miR-203 was downregulated, showing a significantly different expression in cervical SCC, compared to normal epithelium. These dysregulated miRNAs mainly controlled the apoptosis signaling pathways and cell cycle regulation [82].

### 4.7. Breast Cancer

The role of miR- 345 in breast cancer (BRCA) was noted in Pogribny et al., which was an investigation of the cisplatin-resistant acquisition phenotype, where miRNA alterations in the MCF-7 human breast adenocarcinoma cells were studied. They identified a total of 103 miRNAs that were differentially expressed (46 upregulated and 57 downregulated) in MCF-7 cells resistant to cisplatin. The most significantly dysregulated miRNAs were miR-146a, miR-10a, miR-221/222, miR-345, miR-200b, and miR-200c. miR-345 in breast cancer was downregulated, helping in cisplatin-resistant phenotype acquisition. These differentially expressed miRNAs are involved in controlling cell signaling, cell survival, DNA methylation, and invasiveness. Further, they also demonstrated that miR-345 targets the human multidrug resistance-associated protein 1 (ABCC1/MRP1). These suggest that the dysregulated miRNA expression is critically associated with the cisplatin-resistant phenotype [83,84] (Figure 1). Ulasov et al. recently showed possible functional interactions between KISS1, E-cadherin, and miR-345. The tumor microenvironment, which controls breast cancer spreading via miR-345-regulated KISS1 expression, might modulate metastatic spreading via a mechanism involving the upregulation of E-cadherin production [85] (Figure 1). Thus, miR-345 has a predominant role in breast cancer and could be a great therapeutic target.

### 4.8. Oral Carcinoma

The potential role of miRNAs has been well documented in the pathogenesis and progression of oral squamous cell carcinoma (OSCC). The role of miR-345 was initially studied by Cervigne et al., showing 109 miRNAs were highly expressed, exclusively in progressive invasive OSCC. Among the 109 miRNAs, three miRNA (miR-21, miR-181b, and miR-345) expressions were consistently increased and associated with increased lesion severity during progression. Overexpression of these three miRNAs may, therefore, play an important role in the malignant transformation. Further, miR-345 expression was increased, indicating it might play a critical role in the malignant transformation of oral carcinoma. They also observed the upregulated miR-345 had no significant role in pathogenesis of OSCC [86,87]. The recent study by Wu et al. showed contradictory results to the above study’s statement, evidencing that the expression levels of miR-345 were downregulated in OSCC tissues, compared to normal tissue, and highlighting miR-345 as a tumor suppressor in OSCC [88]. The cell proliferation and cell cycle arrest at the G1 phase, exhibited by miR-345, are similarly observed in other cancerous conditions reported. The in silico and in vitro data showed a miR-345 binding site at the 3′UTR of ZEB2, and an inverse correlation between miR-345 and ZEB2 mRNA expression was reported in OSCC [88] (Figure 1). The general role of ZEB2 is as a DNA-binding transcription factor that contributes to a series of cellular functions, such as cell proliferation and colony formation, as well as migration, invasion, and tumor cell-induced angiogenesis [89]. ZEB is also known as a transcriptional repressor of E-cadherin in cancer, thereby facilitating EMT in breast and colon carcinoma. ZEB also helps in tumor angiogenesis via the TGF-β signaling cascade [90,91]. Elevated ZEB2 expression leads to poor outcomes in other cancers like breast cancer, ovarian cancer, oral squamous cell carcinoma, and PC [92,93].

### 4.9. Mesothelioma

Malignant mesothelioma (MM) is an aggressive type of cancer that arises from mesothelial cells, mainly due to former asbestos exposure. Guled et al. demonstrated that miR-345 was shown to be highly expressed in malignant mesothelioma, compared with normal samples (Figure 1). They assessed eleven miRNAs (let-7b*, miR-1228*, miR-195*, miR-30b*, miR-32*, miR-345, miR-483-3p, miR-584, miR-595, miR-615-3p, and miR-885-3p), which were highly expressed. Nine others (let-7e*, miR-144*, miR-203, miR-340*, miR-34a*, miR-423, miR-582, miR-7-1*, and miR-9) were unexpressed or had severely reduced expression levels. These miRNAs target genes are primarily affected in MM, including *CDKN2A*, *NF2*, *JUN*, *HGF*, and *PDGFA* [94].

### 4.10. Acute Myeloid Leukemia

The study conducted by Ying et al. shows that miR- 345 expression was significantly lower in acute myeloid leukemia (AML) patients, when compared to normal patients, helping in AML cell proliferation. AML is a malignant hematopoietic disease, which is caused by abnormalities in the hematopoietic stem cell. Further in silico analysis revealed that AKT1/AKT2 are the targets of miR-345. AKT1/2 was negatively correlated with miR-345-5p expression and was responsible for apoptosis and cell proliferation [95]. They also found out that expression levels of miR-345 were decreased in both in vitro and in vivo; when they overexpressed miR-345, they observed cell cycle arrest at the G0/G1 phase and, thus, reduced cell proliferation. Further, the study identified the PI3K/AKT signaling pathway as a critical pathway for the malignancy, providing new insight into AML treatment, diagnosis, and prognosis for translational research. AKT signaling was previously reported to be crucial in leukemia cell proliferation, and the PI3K/AKT/mTOR pathway is important in AML evolution and relapse [96]. Thus, targeting the AKT signaling pathway may provide a new avenue for leukemia treatment [97]. PI3K activation can phosphorylate its downstream AKT. Further, AKT affects its downstream cascades, such as CREB and mTOR [98].

### 4.11. Anaplastic Thyroid Carcinoma

A study conducted by Marini et al. showed that anaplastic thyroid carcinoma (ATC)-and papillary thyroid carcinoma (PTC)-derived cell lines, along with their tissue samples, showed a differential expression of multiple miRNAs, in which miR-345 showed a significant downregulation [99] (Figure 1)**.**

### 4.12. Lip Cancer

The study conducted by Assao et al., to check expression levels of miR-181b, miR-21, miR-31, and miR-345 in actinic cheilitis with and without epithelial dysplasia and with lower lip squamous cell carcinomas (LLSCC), revealed that increased expression of miR-345, miR-181b, and miR-31 was found in actinic cheilitis without epithelial dysplasia, in comparison with that of actinic cheilitis with epithelial dysplasia and LLSCC (Figure 1). Thus, this miRNA signature can help to identify actinic cheilitis, with the potential to progress to lip cancer [100].

### 4.13. Glioblastoma

A study by Cao et al. on glioblastoma (GBM) suggested that long non-coding RNA (lncRNA) LINC01426 plays an important role in the progression of glioblastoma by downregulating miR-345-3p, thus upregulating VAMP8, which helps in cell proliferation (in silico analysis showed VAMP8 as potential target of miR345-3p). In addition, they also found that, when miR345-3p inhibitor was used and VAMP8 was overexpressed, they could partly rescue the cell proliferation inhibition by knockdown of LINC01426 in U251 GBM cells. Thus, their findings suggested that any of the deregulation in the LINC01426/miR345-3p/VAMP8 axis promotes GBM development [101] (Figure 1). miRNA can interfere with LncRNA and its downstream pathway as ceRNA, affecting its post-transcriptional regulation [102]. Competing endogenous RNAs (ceRNA) regulate other RNA transcripts, by competing for shared miRNA and regulatory networks, and are generally involved in different biological processes of cancer, such as tumorigenesis, EMT, and metastasis cascades [103]. The interaction between lncRNA-miRNA functional networks has been a new area of attraction in recent years. It is still unclear from this study as to whether LINC01426 binds to miRNA to regulate GBM development, and what the mechanisms behind why LINC01426 is elevated in GBM are. Here, we have summaried the role of miR-345 in various malignancies in Table 1.

Figure 1 below summarizes the mechanistic approach of miR-345 in pancreatic cancer, along with various other cancers.

## 5. miRNA Nanodelivery

Despite promising studies showing the potential for the use of miRNA as therapeutics, current clinical applications of miRNA are few, including only two clinical trials. The first is a phase 1 trial using INT 1B3, a lipid nanoparticle formulation of miR-193s-3p, to treat advanced solid tumors [104]. The second is a phase 1 and 2 trial using AMT-130, an adeno-associated viral vector expressing miHTT, to treat Huntington’s disease [105]. Given the lack of phase 3 clinical trials and the broad range of potential applications of miRNA treatments, it remains undetermined as to what the optimal delivery methods will be.

Successful delivery of miRNA to target sites, including PC, presents many challenges. These challenges include stability, transport, diffusion, off-target effects, cellular entry, and endosomal escape [106]. Naked miRNA is easily degradable by nucleases, causing poor stability in the blood-stream and reduced circulation half-life, thus leading to unfavorable pharmacokinetics [107]. Naked miRNA lacks the ability to specifically target a desired cell type or location in the body; this nonspecific targeting results in off-target toxicity and poor bioavailability, as well. The phosphate groups in naked miRNA impart a negative charge, which creates difficulty in penetrating cell membranes, due to electrostatic repulsion. miRNA also degrades in the low pH environments found in endosomes. When the naked miRNA is successfully taken in by target cells via endocytosis, it can be degraded in the endosome, before being released into the cytoplasm.

Viral vectors and nanoparticles can be used as delivery vehicles for miRNA to address these previously mentioned challenges. The main goals of the nanodelivery systems are to protect the miRNA from degradation, efficiently transport the miRNA to the target site, and facilitate cellular uptake and endosomal escape. While viral vectors are generally highly efficient in transduction, the major disadvantage of viral vectors is that they are highly immunogenic [108]. Non-viral vectors, in the form of nanoparticles, are much more biocompatible and are tunable in physicochemical properties. Nanoparticle delivery vehicles with a positive charge can either electrostatically complex or encapsulate the negatively charged miRNA. Electrostatic complexation protects the miRNA from degradation and shields the negative charges. Low encapsulation efficiency is common in the delivery of miRNAs, which is often due to the encapsulation method or biomaterial chosen as the delivery system [109]. Different biomaterials have been investigated for the nanodelivery of miRNA for cancer treatment, including liposomes/lipids [110,111], polymers [112,113], and inorganic nanoparticles [114]. An important aspect of these nanoparticles is their ability to deliver small molecule drugs, in combination with miRNA, to synergistically treat PC and potentially increase the efficacy of the treatment. Additionally, each of these types of nanoparticles can be surface-modified for targeted delivery, which will be discussed later. Liposomes, such as Lipofectamine^TM^, are biocompatible, with generally low toxicity. They are also amphiphilic, which can be an advantage for the combination delivery of drugs and miRNA. Inorganic nanoparticles have the advantage of being highly stable and biologically inert. However, they are not biodegradable and other biomaterials, such as polymers or lipids, are typically needed to allow for the desired chemical attachments or electrostatic complexation with miRNAs. Polymers, on the other hand, are tunable in size, molecular weight, surface charge/groups, and morphology, which controls their biodegradability, biocompatibility, specific targeting, and overall efficiency as nanodelivery systems. Nanoparticles of biodegradable polymers are also good candidates for the sustained release of miRNA and drugs.

Targeting strategies increase the accumulation of the miRNA at the target site. A broad range of biological ligands can be attached to the nanoparticle surface to bind to specific target cell receptors. These ligands could include proteins, antibodies, or polysaccharides, among other biological molecules [115]. Stimuli responsiveness can also be a form of targeting. Differences in the physicochemical properties of the target site, such as pH or hypoxia, in PC and other cancers, can be leveraged to trigger the release of the miRNA from the delivery system. For example, Gupta et al. (2018) developed a pH-responsive, silica-supported mesoporous titania nanocarrier to deliver paclitaxel and miR-708 for colorectal carcinoma, which showed a significantly higher release at low pH [116]. In addition to target site specificity, the rate of miRNA release is important for maintaining a therapeutic concentration at the target site.

The sustained release of therapeutic miRNAs could prevent the need for multiple doses to be administered. The controlled release of miRNA is possible with polymer nanoparticles as the delivery vehicle. Arora et al. developed PLGA-based nanoparticles to deliver miR-150 to PC, which showed a sustained release over 14 days [117]. The rate of release can be tuned by altering the monomers, methods of polymerization, and encapsulation [118]. Changing the size, surface charge, and degree of hydrophobicity are also important in drug delivery for maximizing the circulation time and cellular uptake of the nanoparticle [119]. Hydrophobic nanoparticles are more easily recognized by the reticuloendothelial system and removed from circulation, decreasing the bioavailability [119]. Highly charged nanoparticles have also been shown to lead to removal from circulation and can also exhibit enhanced cytotoxicity [120]. The ideal size for nanoparticles is dependent on the target site [119]. For example, the enhanced permeation and retention effect in tumors can be leveraged by keeping the nanoparticle diameter between 10–100 nm to increase accumulation in the tumor microenvironment [121]. Non-biodegradable nanoparticles need to be under 17 kDa in molecular weight to be cleared by the kidneys and excreted [122].

**Figure 2:**
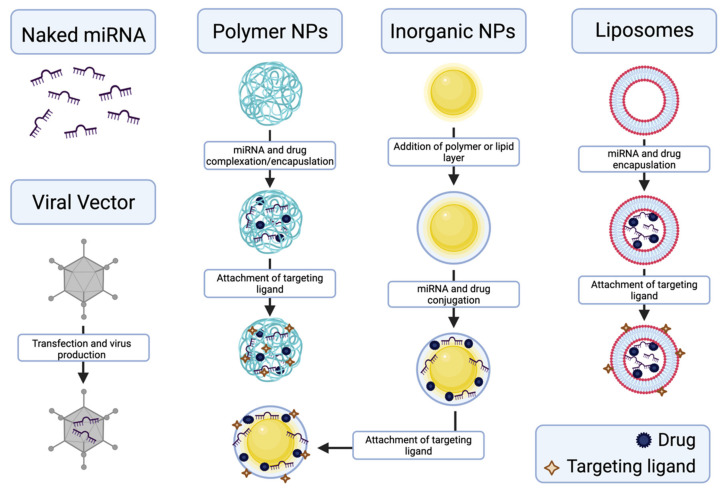
Schematic overview of the production of nanodelivery vehicles, including viral vectors, polymeric nanoparticles, inorganic nanoparticles, and liposomes, for miRNA and drug delivery. Created with BioRender.com.

Few systems have been developed for the co-delivery of miRNA and chemotherapeutic drugs for the treatment of PC. These systems aim to provide a synergistic anticancer effect in treating PC with miRNAs and drugs. Figure 2 summarizes the methods for delivery of miRNA and drugs, using viral vectors and nanoparticles.

miR-205, miR-345, and miRlet-7b have all been used in combination delivery systems for PC treatment. miR-205 and gemcitabine were delivered using a cationic copolymer, poly(ethyleneglycol)-block-poly(2-methyl-2-carboxyl-propylenecarbonate-graft-dodecanol-graft-cationic ligands) (mPEG-b-PCC-g-GEM-g-DC-g-CAT) [113]. A film hydration method was used to form polyplexes containing gemcitabine and miR-205. Significant tumor reduction was seen in the in vivo study on ectopic tumors in athymic nude mice, when treated with these polyplexes [113]. A micellar formulation of poly(ethylene glycol)-block-poly(2-methyl-2-carboxyl-propylene carbonate-graft-dodecanol-graft-tetraethylenepentamine) (PEG-b-PCC-g-DC-g-TEPA) was used to deliver miR-let7b and hedgehog inhibitor GDC-0449 in an orthotopic PC mouse model [123]. These micelles, containing miR-let7b and GDC-0449, were also formed by film hydration. Significant tumor regression was seen in the combined delivery group [123].

For the delivery of miR-345 for PC treatment, we have developed a dual delivery nanoscale device (DDND) that features several of the desired properties for miRNA delivery, mentioned above, and synergistically transports miR-345, as well as gemcitabine to PC cell lines and in vivo mouse models [22]. The DDND is composed of a temperature- and pH-responsive pentablock copolymer, electrostatically complexed with miR-345 and gemcitabine-encapsulated Pluronic F127 micelles. The pentablock copolymer consists of a Pluronic F127 middle block (poly(ethyleneoxide)-block-poly(propyleneoxide)-block poly(ethyleneoxide) (PEO-PPO-PEO)) with poly (2-diethylaminoethyl methacrylate (PDEAEM) end blocks. The Pluronic F127 middle blocks are temperature-responsive, while the PDEAEM end blocks are cationic and pH-responsive. The cationic PDEAEM end blocks allow for electrostatic complexation of miR-345. The temperature-responsive middle blocks allow for temperature-dependent gelation at near body temperatures, allowing for a gene and drug depot and sustained release. The pH sensitivity of the polymer is tuned to enable endosomal escape easily in cancer cells but not in normal cells, allowing for the targeting of cancer cells [124]. These DDND showed very high encapsulation efficiencies of miRNA and good serum and RNase stability. Increased transfection efficiency was also seen, compared to the commercially available transfection agent, Lipofectamine^TM^ [22].

## 6. Conclusions

This review cultivates the hypothesis on how miR-345 plays a vital role as a prognostic and therapeutic target and surveys effective ways to deliver to the targeted sites in PDAC and various other cancer types. The limitations of current treatment strategies in PC reinforce the need for new avenues of research to be explored, in order to achieve potential breakthroughs. Significant gaps remain in the understanding of this disease; although the treatment options are continually evolving, they still have had limited success. There has been a recent drive to fund large consortia and specialist research into pancreatic ductal adenocarcinoma, but there is much work to be done to enable similar breakthroughs, as seen for other cancer types. In this review, we emphasized the role of miR-345 in different cancers, with a particular interest in PDAC and its effective miRNA nanodelivery for successful therapeutic application, as miRNA delivery will be the potential key for the successful treatment of cancers in the future.

## Figures and Tables

**Table 1:** Denotes the role of MiRNA-345 in different cancers.

Section No.	Cancer Type	miR345 Functional Role(s)	miR345 Target(s)	References
1	Pancreatic (PC, PDAC)	Disruption of mitochondrial membrane potential, resulting in caspase-dependent apoptosis. Mediation of anti-apoptotic BCL2 in caspase-independent apoptosis. Overexpression evidenced increased gemcitabine cytotoxicity, increased epithelial markers, and decreased mesenchymal markers. Inactivation of NFkB signaling by targeting CCL8 and downstream inflammation response. Downregulation of MUC4 and SHH impacting EMT.	BCL2 CCL8	[41,42,49]
2	Colon and Rectal (CRC, LARC)	Strong prognostic marker for overall survival and response to cetuximab/irinotecan treatment in mCRC. Implicated as methylation-sensitive miRNA, with a possible role in CRC growth inhibition. High expression associated with chemoradiotherapy resistance.	BAG3	[37,59,60]
3	Gastric (GC)	Low expression implicated in lymph metastasis, advanced GC stage, reduced overall, and disease-free survival. Knockdown evidenced aggressive phenotype and EMT. Prevents tumor metastasis and EMT by targeting FOXQ1.	FOXQ1	[63]
4	Prostate (PCa, CRPC)	A diagnostic marker for PCa by circulating miR-345-5p levels. Inhibition of tumor cell cycle and eventual tumor suppression, through CDKN1A-mediated cyclin/CDK complex inhibition. Tumor progression inhibition via Smad1 targeting.	CDKN1A Smad1	[68,69]
5	Lung (NSCLC)	Inhibition of pro-tumoral YAP1 and downstream prevention of tumor invasion and metastasis. Low expression associated with reduced overall survival, malignant clinical parameters, and poor prognosis.	YAP1	[70,71]
6	Liver (HCC)	High expression implicated in higher survival rates. Knockdown led to EMT through IRF1-mediated mTOR/STAT3/AKT signaling and downstream targeting of slug, snail, and twist Evidenced apoptotic inhibition in Huh7 cells through p21 targeting. Upregulation of HBV replication levels, leading to HCC cell proliferation. YAP1 and YAP/TAZ-related role in EMT, tumor progression and metastasis. Knockdown evidenced more significant HCC lung metastasis.	p21 IRF1 YAP	[40,73,74,75,78]
7	Cervical (CC, CIN)	Upregulation evidenced in CIN versus normal epithelium. Associated with apoptotic signaling and cell cycle regulation.	N/A	[82]
8	Breast (BRCA)	Downregulation associated with cisplatin-resistant phenotype, possibly via targeted ABCC1. Implicated in brain metastasis by KISS1 and E-cadherin signaling.	ABCC1 KISS1	[83,84,85]
9	Oral (OSCC)	High expression associated with increased lesion severity and progressive invasive OSCC phenotype. Downregulated in OSCC tissue versus normal samples. Implicated in ZEB2 suppression through an inverse expression relationship.	ZEB2	[86,87,88]
10	Mesothelioma (MM)	1. Upregulation evidenced in MM tissue versus normal samples.	N/A	[94]
11	Acute Myeloid Leukemia (AML)	Implicated in cell proliferation and apoptosis via AKT1/AKT2 targeting. Overexpression induced G0/G1 arrest, leading to decreased tumor proliferation. Downregulated in AML versus normal samples.	AKT1/ AKT2	[95]
12	Anaplastic Thyroid Carcinoma (ATC)	1. Downregulated in ATC tissues versus normal samples.	N/A	[99]
13	Lip Cancer (LLSCC)	1. Downregulation evidenced in LLSCC versus actinic cheilitis samples.	N/A	[100]
14	Glioblastoma (GBM)	Downregulated in GBM versus normal samples Implicated in regulation of VAMP8, a cell proliferation-facilitating protein.	VAMP8	[101]

## Data Availability

As this is a review article, this section is not applicable.

## Abbreviations

PC, Pancreatic Cancer; PDAC, Pancreatic Ductal Adenocarcinoma; NETs, Pancreatic Neuroendocrine Tumors; DM, Diabetes Mellitus; CA 19-9, Carbohydrate Antigen 19-9; GC, Gastric Cancer; CRC, Colorectal Carcinoma; PCa, Prostate Cancer; BRCA, Breast Cancer; GEM, Gemcitabine; NLS, Nuclear Localization Signal; EMT, Epithelial-Mesenchymal Transition; mCRC, Metastatic Colorectal Cancer; OS, Overall Survival; PFS, Progression-Free Survival; pre-CRT, Preoperative Chemoradiotherapy; LARC, Locally Advanced Rectal Cancer; DFS, Disease-Free Survival; CRPC, Castration-Resistant Pancreatic Cancer; NSCLC, Non-Small-Cell Lung Carcinoma; HCC, Hepatocellular Carcinoma; OSCC, Oral Squamous Cell Carcinoma; MM, Malignant Mesothelioma; AML, Acute Myeloid Leukemia; ATC, Anaplastic Thyroid Carcinoma; PTC, Papillary Thyroid Carcinoma; LLSCC, Lower Lip Squamous Cell Carcinoma; DDND, Dual-Delivery Nanoscale Device.
